# Biology of fowl adenovirus type 1 infection of heterologous cells

**DOI:** 10.1007/s00705-012-1413-9

**Published:** 2012-07-20

**Authors:** Satoshi Taharaguchi, Rina Fukazawa, Miho Kitazume, Hayato Harima, Kensuke Taira, Kenji Oonaka, Motonobu Hara

**Affiliations:** 1 Department of Microbiology II, School of Veterinary Medicine, Azabu University, Kanagawa, 252-5201 Japan; 2 Department of Microbiology, School of Life and Environmental Science, Azabu University, Kanagawa, 252-5201 Japan; 3 Department of Parasitology, School of Veterinary Medicine, Azabu University, Kanagawa, 252-5201 Japan

**Keywords:** Fowl adenovirus, Abortive infection

## Abstract

The JM1/1 strain of fowl adenovirus (FAV) serotype 1 isolated from gizzard erosion was used to investigate the biology of FAV in homologous (susceptible) and heterologous cells. The FAV JM1/1 strain is capable of efficient multiplication in primary chicken kidney (CK) cells, but not in Crandell-Rees feline kidney (CRFK) cells or Vero cells. FAV adsorption in heterologous cells was slightly higher than in CK cells. An early gene encoding a DNA-binding protein and a late gene encoding the hexon protein were expressed in CK cells. Only the early gene was expressed in Vero cells. Neither of these genes was expressed in CRFK cells. These results suggest that the virus was unable to multiply effectively due to suppression of viral gene expression in the heterologous cells used in this study.

The family *Adenoviridae* includes five genera: *Mastadenovirus*, *Aviadenovirus*, *Atadenovirus*, *Siadenovirus*, and *Ichtadenovirus*. No antigenicity is shared by members of these genera [2]. The adenoviruses contain at least 10 structural proteins. Soluble antigens contain various genus-, subgenus-, and type-specific epitopes [5]. The soluble antigens form icosahedral particles (70-90 nm in diameter) composed of 252 capsomeres, with a single linear double-stranded DNA molecule as the genome. The capsomeres are composed of 12 pentons and 240 hexons. The pentons have one or two fibers [17].

Of the three genera whose members infect birds, avian adenoviruses include fowl adenoviruses (FAVs), goose adenovirus, and duck adenovirus. These adenoviruses are prevalent among birds. The FAVs are capable of efficient multiplication in chicken kidney (CK) cells, and chicken embryonic liver cells show typical cytopathic effects [3].

FAV serotype l, called chick embryo lethal orphan (CELO) virus, transduces human lung, liver and kidney cells for gene transfer applications [6, 19]. Earlier investigations were intended not to analyze FAV pathogenicity but to use FAV as a vector. To identify factors that allow FAV to acquire pathogenicity, abortive infection of FAV was investigated. In this study, we have infected heterologous cells, Crandell-Rees feline kidney (CRFK) and Vero cells, with the FAV JM1/1 strain to examine its biology.

The JM1/1 strain (group I serotype 1), originally isolated from an outbreak of avian gizzard erosion in a commercial broiler flock in Japan, was used [21]. The strain was propagated in CK cells, Vero cells, and CRFK cells, which were grown on monolayer cultures in MEM supplemented with 5 % fetal bovine serum. Cells were incubated at 37 °C with 5 % CO_2_. Multiplication of FAV in homologous or heterologous cells was compared. Cells were plated on 60-mm tissue culture dishes. FAV JM1/1 was used to inoculate each dish at a multiplicity of infection (MOI) of 1. After allowing adsorption for 1 h, the cells were washed three times with PBS. The cell culture medium was harvested at the specified times. A virus assay was done by inoculating CK cell monolayers in 24-well plastic plates with various sample dilutions (0.1 mL per well) and observing them for any cytopathogenic effects. The virus concentration was calculated using the Reed-Muench formula [13] and reported as TCID_50_ (50 % tissue culture infectious dose) using four wells per dilution. Virus replication in CK cells were detected at 24 h and continued until 48 h after inoculation. On the other hand, replication of FAV JM1/1 in Vero and CRFK cells was not detected at any of the time points (Fig. 1a). In the tested heterologous cells, infection was abortive.

**Fig. 1:**
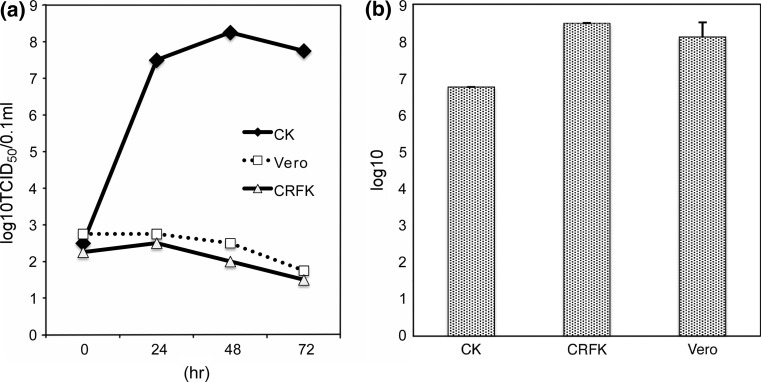
(**a**) Susceptibility of cells to FAV JM1/1 infection. Cells were infected at an MOI of 1. The virus present in cell culture supernatants at 0, 24, 48, and 72 h p.i. was titrated in CK cells. (**b**) FAV binding assay by real-time PCR Binding assay carried out using cells with FAV JM1/1. The mean values and standard derivations represent three independent assays

To examine FAV JM1/1 adsorption to CK, CRFK, and Vero cells, cells were distributed to 12-well plates and grown to confluence. Prior to inoculation, cells were washed twice with PBS. The inoculum (MOI = 1) was incubated with the cells for 2 h at 4 °C. Then, cells were washed three times with the culture medium. DNA was extracted using a DNeasy Blood and Tissue Kit (QIAGEN) according to the manufacturer’s manual. The cell-associated viral DNA was quantified by a real-time PCR method using DBP3’ (5’- ACC TCG TAC CGT GGA GTT - 3’) and DBP5’ (5’- GGT AAA GCG CCT TCG TCC AGT - 3’) primer pairs. For each experiment, three independent assays were performed. Almost the same amount of FAV DNA as in susceptible CK cells could be detected in Vero and CRFK cells by real-time PCR. These results suggest that FAV may be adsorbed onto non-susceptible Vero and CRFK cells as well as susceptible CK cells (Fig. 1b).

To demonstrate FAV JM1/1 infection and protein expression in cells, anti-FAV chicken serum and anti-hexon monoclonal antibody (mAb) [18] were used for detection using a fluorescent antibody technique. Cells in 24-well culture plates with a glass cover slip were infected with FAV at an MOI of 1 at 37 °C. After 24 and 48 h incubation, the infected cells were fixed with 4 % paraformaldehyde in PBS for 30 min at room temperature. The infected cells were then washed once with PBS and permeabilized with 0.05 % Triton X-100 in PBS for 30 min at room temperature. Blocking at room temperature in PBS containing 1 % skim milk was performed for 30 min. The infected cells were then incubated with chicken anti-FAV antibody or anti-hexon mAb diluted in PBS containing 1 % skim milk for 60 min at 37 °C, washed with PBS, and then incubated with FITC-labeled goat anti-chicken or mouse IgG diluted in PBS containing 1 % skim milk for 60 min at 37 °C. The samples were washed three times with PBS and examined for fluorescence. FAV-specific protein expression was detected in FAV-infected CK cells at the 24-h mark using anti-FAV chicken serum. No FAV-specific protein expression was detected in FAV-infected CRFK and Vero cells even after a 72-h incubation (Fig. 2a). Hexon protein expression was detected in FAV-infected CK cells at 24 h or later using anti-hexon mAb. However, hexon protein expression was not detected in FAV-infected CRFK or Vero cells (Fig. 2b). Neither the late protein nor proteins detectable with anti-FAV chicken serum could be detected in FAV-infected CRFK and Vero cells.

**Fig. 2:**
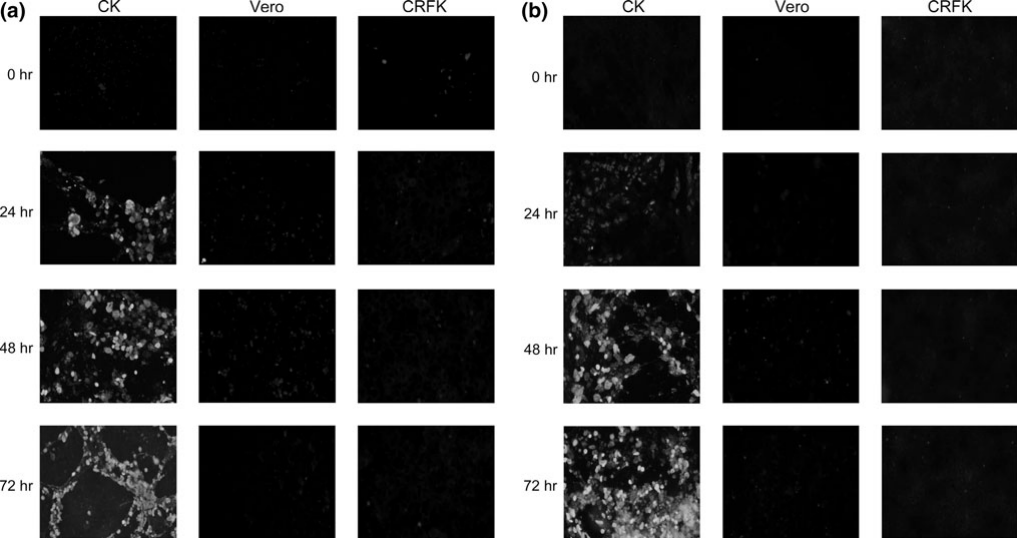
Fluorescent assay of FAV JM1/1-infected cells. Confluent monolayers of indicated cells were infected with FAV JM1/1 (MOI = 1). At 0, 24, 48, and 72 h p.i., cells were stained with (**a**) anti FAV JM1/1 chicken polyclonal antibody and FITC-conjugated anti-chicken IgG or (**b**) anti-hexon mAb and FITC-conjugated anti-mouse IgG

To examine the expression of mRNA of an early gene encoding DNA-binding protein (DBP) and a late gene encoding the hexon protein in FAV JM1/1-infected CK, CRFK, and Vero cells, RNA was isolated from infected cells after 0, 24, and 48 h incubation periods using Tripure Isolation Reagent (Roche). cDNA was synthesized from purified DNase-I-treated total RNA using MMLV reverse transcriptase and random primers (Invitrogen, USA) at 30 °C for 10 min and 40 °C for 60 min according to the manufacturer’s protocol. For DBP, hexon, and β-actin transcript amplification, the primers DBP5’ and DBP3’, Hexon5’ (5’- ACT ACA CTC AGA CCC TGA GTT A - 3’) and Hexon3’ (5’- CTC GGA GTT GAG CGT TC - 3’), and ActinF (5’- AAC GAG CGG TTC CGC TGC CC - 3’) and ActinR (5’- GAT CTT GAT CTT CAT CGT GC - 3’) were used, respectively. DBP and hexon mRNA expression was detected in CK cells at 12 and 24 h after FAV JM1/1 infection. DBP expression was detected in Vero cells at 12 and 24 h after FAV JM1/1 infection, but no hexon mRNA expression was detected at these time points. Neither DBP nor hexon expression was observed in FAV-infected CRFK cells (Fig. 3).

**Fig. 3:**
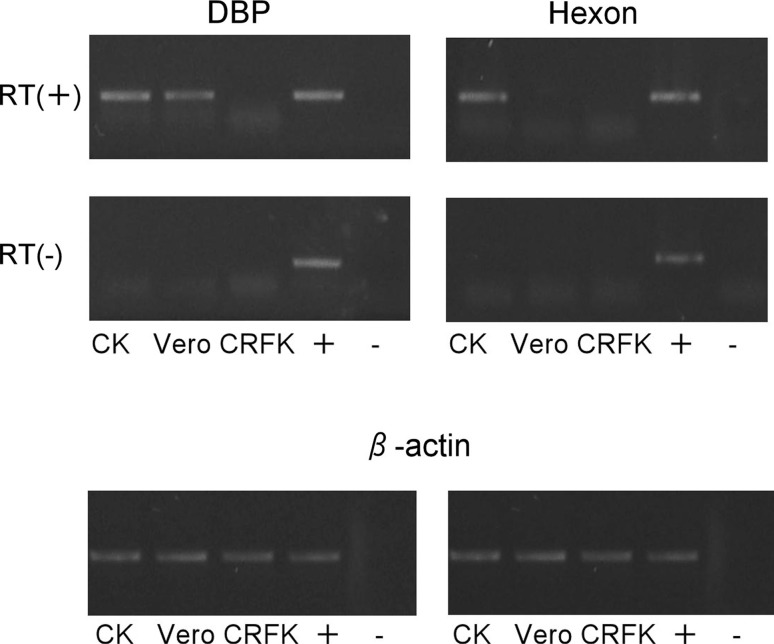
FAV transcript analysis by RT-PCR. Cells were infected at an MOI of 1, and RNA was isolated at 24 h. RT-PCR was performed with 1 μg of total RNA using primers specific for DBP, hexon, and β-actin transcripts

The biology of FAV JM1/1 isolated from gizzard erosion was examined in CK cells and heterologous cells. FAV JM1/1 was adsorbed onto the surface of susceptible CK cells (permissive cells) to express an early gene encoding DBP and a late gene encoding the hexon protein in order to produce viral proteins and release infectious viruses. FAV was adsorbed onto the surface of Vero cells to express the early gene, DBP, but not the late gene, hexon. No proteins could be detected with anti-FAV chicken serum. Thus, Vero cells express FAV receptors and transcribe the early gene, DBP, after adsorption and penetration. HAVd2 infection suppresses multiplication in simian cells by inhibiting splicing and transport [14]. FAV might also have suppressed multiplication by inhibiting splicing and transport.

FAV was adsorbed into the surface of CRFK cells but induced neither DBP nor hexon gene expression. No DBP expression was detected in CRFK cells, suggesting possible inhibition during endocytosis. FAV was adsorbed onto Vero and CRFK cells. Thus, receptors for FAV JM1/1 may exist on these cells.

The CELO strain of FAV serotype 1 has long and short fibers. The long fiber (fiber-1) is essential for transduction through the coxsackie virus and adenovirus receptor (CAR). The short fiber (fiber-2) is critical for infection of chicken cells [19]. The CAR-dependent adenovirus should bind to CAR and to the integrin αυβ3 or αυβ5 for infection and multiplication [20]. Human adenoviruses are classified into subgroups A-F. The pathogenicity and other infectious factors of adenovirus type 5 that belong to subgroup C have been investigated in detail. Adenovirus type 5 and many other adenoviruses utilize CAR for infection. Adenoviruses that belong to subgroup B may recognize molecules other than CAR for infection. However, the molecules remain unidentified [15, 16]. The FAV CELO parent strain transduce human and mouse cell lines. However, fiber-1-deficient mutant strains do not transduce CAR-dependent cells [19]. To examine the tropism of measles virus, increasing transcription and budding facilitates viral survival in cells when no efficient receptors, such as SLAM, can be used. Unknown receptors with low entry efficiency can be employed to infect cells [11]. Thus, FAV may use fiber-2 to infect cells using receptors other than CAR, which are expressed in many cells.

The heterologous cells used in this study showed almost the same amount of FAV binding as the susceptible cell line CK but did not allow FAV multiplication. Human cells completely support the infection cycle of human adenovirus, while simian and rodent cells do not. Regarding human adenovirus infection in simian and rodent cells, the host range is limited by reactions between cells and viral components as well as by receptor binding [4]. FAV features low pathogenicity under normal conditions and frequently causes an inapparent infection. Some FAV strains cause hydro-pericardium syndrome, gizzard erosion, pancreatic atrophy and necrosis, and respiratory disorders [7–10]. FAV isolated from the lesions of hydro-pericardium syndrome, gizzard erosion, and pancreatic necrosis could reproduce these lesions, suggesting their high pathogenicity [1, 7, 8, 12, 21]. How FAV JM1/1 causes gizzard erosion may be revealed by identifying intracellular factors that influence FAV multiplication.

## References

[1] Balamurugan V, Kataria JM, Kataria RS, Verma KC, Nanthakumar T (2002). Characterization of fowl adenovirus serotype-4 associated with hydropericardium syndrome in chicken. Comp Immunol Microbiol Infect Dis.

[2] Buchen-Osmond C (2003) Adenoviridae. ICTVdB—The Universal Virus Database, version 3. The Earth Institute, Biosphere 2 Center, Columbia University, AZ, USA

[3] Gelderblom H, Maichle-Lauppe I (1982). The fibers of fowl adenoviruses. Arch Virol.

[4] Lucher LA (1995). Abortive adenovirus infection and host range determinants. Curr Top Microbiol Immunol.

[5] McFerran JB, Adair B, Connor TJ (1975). Adenoviral antigens (CELO, QBV, GAL). Am J Vet Res.

[6] Michou AI, Lehrmann H, Saltik M, Cotten M (1999). Mutational analysis of the avian adenovirus CELO, which provides a basis for gene delivery vectors. J Virol.

[7] Nakamura K, Mase M, Yamaguchi S, Shibahara T, Yuasa N (1999). Pathologic study of specific-pathogen-free chicks and hens inoculated with adenovirus isolated from hydropericardium syndrome. Avian Dis.

[8] Nakamura K, Mase M, Yamaguchi S, Yuasa N (2000). Induction of hydropericardium in one-day-old specific-pathogen-free chicks by adenoviruses from inclusion body hepatitis. Avian Dis.

[9] Nakamura K, Ohyama T, Yamada M, Abe T, Tanaka H, Mase M (2002). Experimental gizzard erosions in specific-pathogen-free chicks by serotype 1 group I avian adenoviruses from broilers. Avian Dis.

[10] Nakamura K, Tanaka H, Mase M, Imada T, Yamada M (2002). Pancreatic necrosis and ventricular erosion in adenovirus-associated hydropericardium syndrome of broilers. Vet Pathol.

[11] Ohno S, Yanagi Y (2006). Cellular tropism and adaptation of the measles virus. Uirusu.

[12] Okuda Y, Ono M, Yazawa S, Imai Y, Shibata I, Sato S (2001). Pathogenicity of serotype 1 fowl adenovirus in commercial broiler chickens. Avian Dis.

[13] Reed LJ, Muench H (1938). A simple method of estimating fifty percent endpoints. Am J Hygiene.

[14] Ross D, Ziff E (1994). Defective processing of human adenovirus 2 late transcription unit mRNAs during abortive infections in monkey cells. Virology.

[15] Sakurai F, Mizuguchi H, Hayakawa T (2003). Efficient gene transfer into human CD34+ cells by an adenovirus type 35 vector. Gene Ther.

[16] Sakurai F, Mizuguchi H, Yamaguchi T, Hayakawa T (2003). Characterization of in vitro and in vivo gene transfer properties of adenovirus serotype 35 vector. Mol Ther.

[17] Shenk T (1996). Adenoviridae: the viruses and their replication.

[18] Taharaguchi S, Ito H, Ohta H, Takase K (2006). Characterization of monoclonal antibodies against fowl adenovirus serotype 1 (FAV1) isolated from gizzard erosion. Avian Dis.

[19] Tan PK, Michou AI, Bergelson JM, Cotten M (2001). Defining CAR as a cellular receptor for the avian adenovirus CELO using a genetic analysis of the two viral fibre proteins. J Gen Virol.

[20] Wickham TJ, Mathias P, Cheresh DA, Nemerow GR (1993). Integrins alpha v beta 3 and alpha v beta 5 promote adenovirus internalization but not virus attachment. Cell.

[21] Yamada K, Takase K, Taharaguchi S, Yamazaki K, Ohta H, Taira K, Takae Y (2005). Fowl Adenovirus isolated from gizzard erosion of broiler: serotype and pathogenicity. Bull Fac Agri Kagoshima Univ.

